# Change in basilar artery length and bending according to aging and vertebral artery dominance: A longitudinal study

**DOI:** 10.1038/s41598-020-65682-x

**Published:** 2020-06-01

**Authors:** Minh Tri Ngo, Hyo Sung Kwak, Gyung Ho Chung

**Affiliations:** Department of Radiology and Research Institute of Clinical Medicine of Chonbuk National University-Biomedical Research Institute of Chonbuk National University Hospital, Jeonju, Korea

**Keywords:** Neuroscience, Anatomy, Magnetic resonance imaging, Ageing, Cerebrovascular disorders

## Abstract

This study aimed to investigate the basilar artery (BA) geometric changes in a longitudinal study. 154 subjects with normal vertebrobasilar arterial systems on magnetic resonance angiography were assigned into two groups: 1) non-dominant vertebral artery (VA) and 2) VA dominance. We defined the dominant VA as either that the VA is 3 millimeters larger in diameter or the VA is connected to BA in a more straight angle. BA imaging was segmented to obtain BA bending length (BABL) and BA length (BAL). A mixed model ANOVA was conducted to investigate the impact of aging and VA dominance on the change of BABL and BAL after 123.6 ± 16.2 months. There was a significant main effect of VA dominance on the change of BABL after about 10 years, F (1,152) = 39.78, p < 0.01. On the other hand, there was a significant main effect of aging on the change of BAL during the same period of time, F (1,152) = 6.64, p = 0.01. Most subjects had an opposite directional relationship between the dominant VA and BA bending (71.3%; p < 0.01). Our study supported the hypothesis that the bending of the BA depends on the dominance of the VA, whereas the increased length of the BA depends on aging.

## Introduction

The basilar artery (BA), which receives blood from 2 vertebral arteries (VAs), provides blood to the posterior circulation of the human brain. The posterior cerebral circulation has less sympathetic innervation than the anterior cerebral circulation^1^, resulting in less trophic support on the arterial wall, which in turn can make the posterior vessels more vulnerable to deformation in structure or morphology when exposed to increases in pressure and blood flow^2,3^. Studies have shown that the elongation and tortuosity of the vertebrobasilar arterial system, have a high degree of variability in presentation^4^, but the causes of these variations have not been determined; aging and hemodynamic factors have been postulated as responsible^5^, but are unproven.

With aging, vascular smooth muscle cells undergo functional changes that alter the normal structure of the vessel wall, predisposing it to the morphological remodeling to minimize their impact on the arterial wall. Many studies have reported changes in arteries during aging, including the carotid artery, coronary artery, aortic artery, and pulmonary artery^6–8^. However, there are few reports on relationships between changes in BA and normal aging. Most data come from studies designed to compare BA geometry differences in young and elderly people rather than from studies of individual persons over time^9,10^. Hence, the relationship between these geometric changes and aging remains unelucidated.

The two VAs are usually asymmetric in size. The difference in the diameter of the VAs more than 3 millimeters (mm), or the asymmetry in the merging of the two VAs to the vertebrobasilar junction, were accepted as criteria for determining the VA dominance^11–13^. Previous studies have also suggested that VA dominance affects BA geometry^12,13^. The unequal VA flow results in changes of the flow force distribution, that influenced the morphological deformation in the VBA, including the basilar artery bending length (BABL) and the basilar artery length (BAL)^13–15^.

To support these hypotheses, we divided the study population into 2 groups (VBA systems with non-dominant VA, and those with VA dominance) and investigate the impact of aging and VA dominance on the change of BA geometry after 123.6 ± 16.2 months.

## Materials and Methods

### Study population

The sampling frame was the database of Chonbuk National University Hospital. From 2004 to 2007, patients who underwent brain magnetic resonance angiography (MRA) at our clinic—were recruited to a longitudinal study, with more than 900 subjects responding to the baseline surveys. Since the baseline MRI, all the subsequent brain MRIs of patients were collected. This paper uses data from 567 patients, who were performed MRA at baseline (2004 to 2007) and after about 10 years (2014 to 2018). The MRAs were performed for clinical purposes.

We investigated the geometry of the VBA system at baseline and after about 10 years. The inclusion criteria were: 1) VBA systems without stenosis and 2) absence of vasculitis of the intracranial arteries, as determined on MRA. Since the posterior cerebral arteries (PCAs) are the terminal branches of the BA, unilateral or bilateral hypoplasia of the PCA (fetal origin of the PCA) may affect BA geometry. Therefore, we excluded VBA systems that had anatomic variants in the PCA to better assess the hemodynamic effect of VAs on change of BA geometry over time. For cases with hypoplasia of the P1 segment of the PCA^16^ or the intracranial part (V4 segment) of VA, the vertebrobasilar junction point and the point of division of the BA could not be determined by the MIMICS (Materialise, Leuven, Belgium) software. Because the anatomy of these cases had to be assessed subjectively, which could have introduced errors in inter-individual BAL calculation, these data were excluded. V4 segment hypoplasia was defined by a diameter of ≤2 mm^17^. Subjects with anatomic anomalies including fenestration of BA, trigeminal artery, or vertebrobasilar dolichoectasia also were excluded.

### MR imaging protocol

The geometry of the VBA system was evaluated with three-dimensional (3D) time of flight (TOF) angiography. The baseline 3D TOF MRA parameters were as follows: ratio of repetition time (TR) to time to echo (TE) = 22/3.9 milliseconds (ms); flip angle = 18°; slice thickness = 0.50 mm; matrix size = 448 × 284; sensitivity encoding (SENSE) factor = 2.5; field of view = 195 × 215 mm; echo train length = 1; number of average (NEX) = 1; and acquisition time = 4 minutes. The scan parameters used for the follow-up were as follow: ratio of TR to TE = 23–25/3.45 ms; flip angle = 20°; slice thickness = 0.90 mm; matrix size = 488 × 249; SENSE factor = 2; field of view = 200 × 200 mm; NEX = 1. The TOF MRA scan time was on average 5.46 minutes.

Image quality per artery was rated on a 4-point scale (1 = poor, 2= moderate, 3 = good, 4 = excellent^18^). Images with a quality <2 points were excluded from the study. VBA systems beyond the field of view were excluded.

### Imaging reconstruction

Figure 2 presents the imaging reconstruction process. Brain MRA was performed uniformly for all subjects. The BA was segmented with MIMICS semi-automated software, using axial-source data of 3D TOF MRA of cerebral arteries^19^. The 3D geometry of the arterial tree was computed from the skeleton of the VBA system with the segmentation tool. The centerlines of the vessels were generated from the VAs to the BA and the PCAs.

### Criteria of vertebral artery dominance

VA dominance was defined as having dissimilar-sized VAs with a difference ≥0.3 mm from side-to-side diameter, or as existing asymmetry in the merging of the two VAs at the vertebrobasilar junction^11–13^. MIMICS software was used to calculate the VA diameters. The vertebrobasilar junction point was determined and served as the origin, from which diameter measurements of both VAs were taken uniformly at 3 mm^20^, based on the vertebrobasilar junction. Using a bilateral comparison of VA diameter and the criterion of angle on the 3D imaging reconstruction, the study population was assigned to 1 of 2 groups: 1) patients with VBA systems with non-dominant VA, and 2) patients with VA dominance.

### Measurement of basilar artery length and basilar artery bending length

A radiologist was presented with the baseline and follow-up TOF images in random order and was blinded to information about when the images were obtained. BAL and BABL were measured as suggested by Nishikata *et al*.^9^. The BAL was measured as a linear distance from the vertebrobasilar junction to the point of the BA division. BABL refers to the distance between the BA bending point and the BA standard line, measured as the vertical distance from the BA standard line to the center of maximum BA bending. The morphological changes of BA encountered were C-shaped deformation, J-shaped, S-shaped, and diagonal line-shaped (BAs that were obliquely inclined across the midline). In the case of S-shaped deformation (multiple bends), the BABL was measured at the closest bend to the vertebrobasilar junction. To improve our quantitative analysis, we modified this method by using the MIMICS software, as illustrated in Fig. 3.

### Statistical analysis

Differences between groups were analyzed using the independent *t*-test or chi-square test, as appropriate for continuous and categorical variables. We performed the mixed model analysis of variance (mixed model ANOVA) to investigate the impact of aging and VA dominance on the change of BA geometry over time. A chi-square test was performed to examine whether there is a significant association of the directional relationship between the dominant VA and BA bending. All analyses were performed with SPSS version 22.0 (IBM Corp, Armonk, NY). Levels of significance were set at 0.05.

### Ethical approval

The protocol of this study was conducted in accordance with the Declaration of Helsinki and was approved by our institutional review board.

### Informed consent

Informed consent was obtained from all participants.

## Results

**Figure 1:**
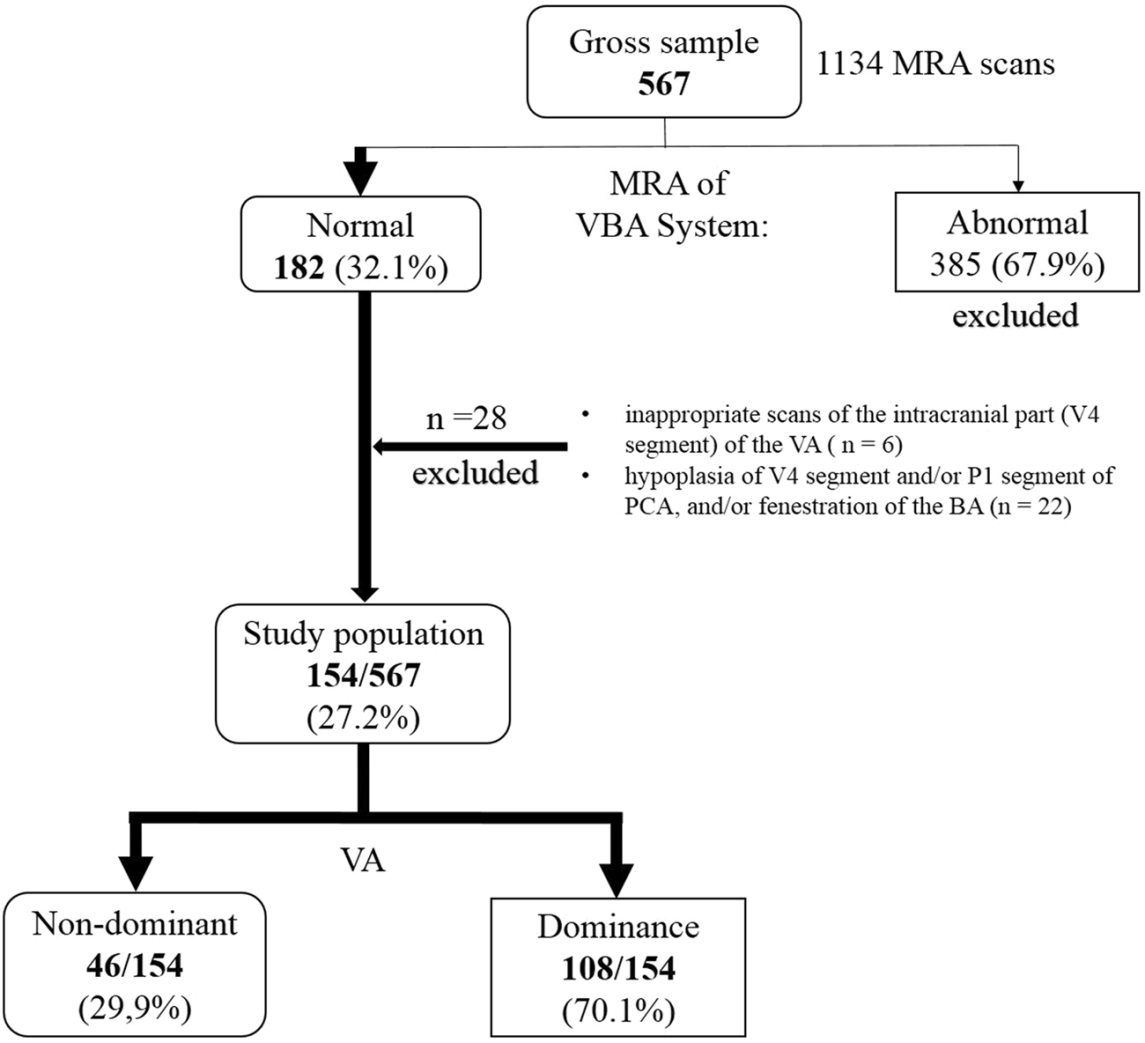
Outline of sample selection. *BA* basilar artery, *MRA* magnetic resonance angiography, *PCA* posterior cerebral artery, *VA* vertebral artery, *VBA system* vertebrobasilar arterial system.

The method of patient sample selection is illustrated in Fig. 1. Of 567 patients initially identified, 182 had a normal MRA of the VBA system. Twenty-eight of these subjects were excluded because of inappropriate scans of the distal portion (V4) of the VA (n = 6), hypoplasia of VAs and/or PCAs, and/or fenestration of the BA (n = 22). Thus, 154 of the 567 patients (27.2%) were enrolled and evaluated (63 men, aged 33–86 years). The subjects were assigned to either VBA system with non-dominant VA (46/154 subjects, 29.9%) or VBA system with VA dominance (108/154 subjects, 70.1%).

**Figure 2:**
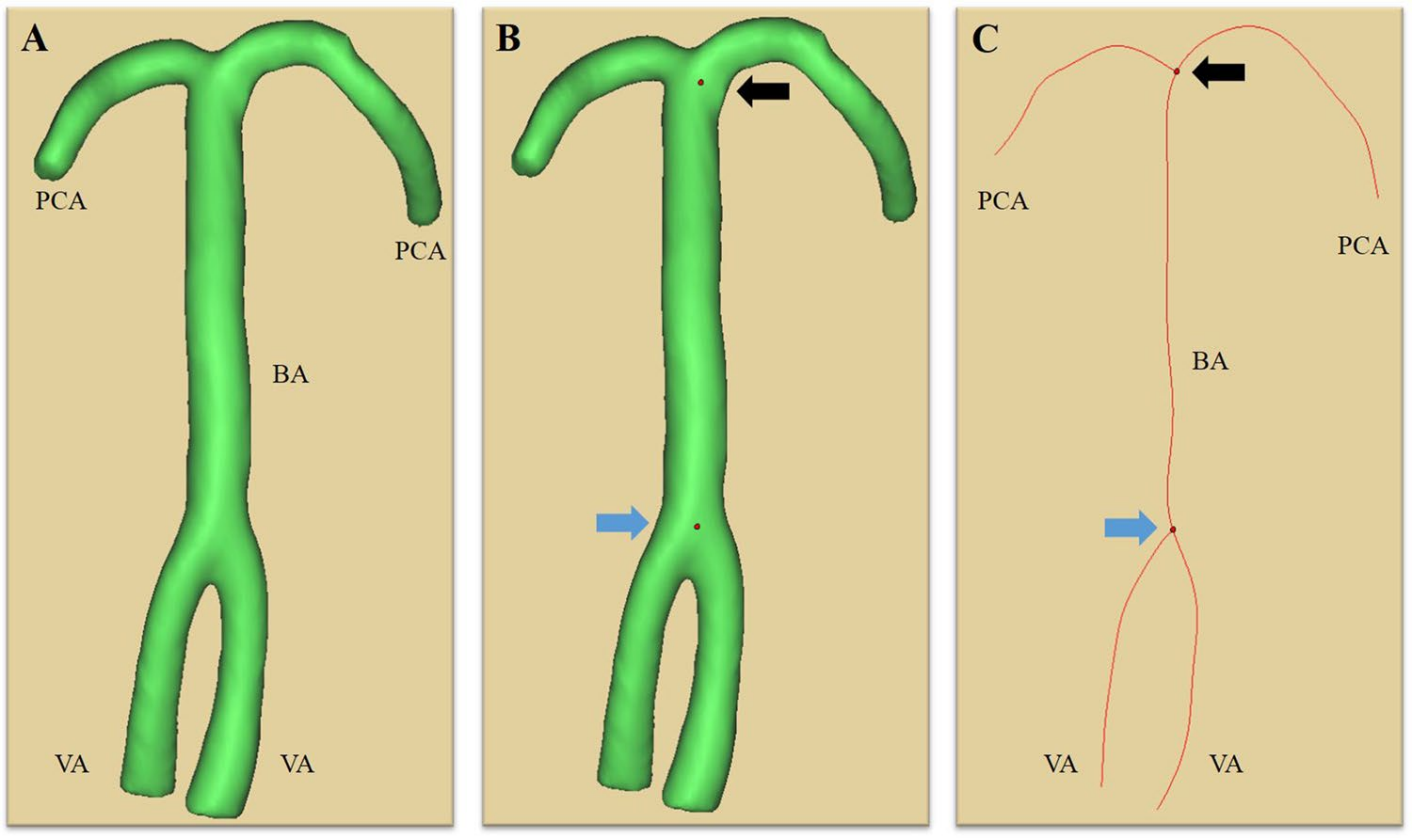
The 3-dimensional geometric characteristics of the arterial tree **(A)** are generated from the skeleton of the vertebrobasilar arterial system by the segmentation software (MIMICS, Materialise, Leuven, Belgium). The vertebrobasilar junction point (blue arrow) and the point of division of the BA (black arrow) are determined **(B)** and the centerlines **(C)** are generated from the vertebral arteries (VAs) to the basilar artery (BA) and the posterior cerebral arteries (PCAs).

**Figure 3:**
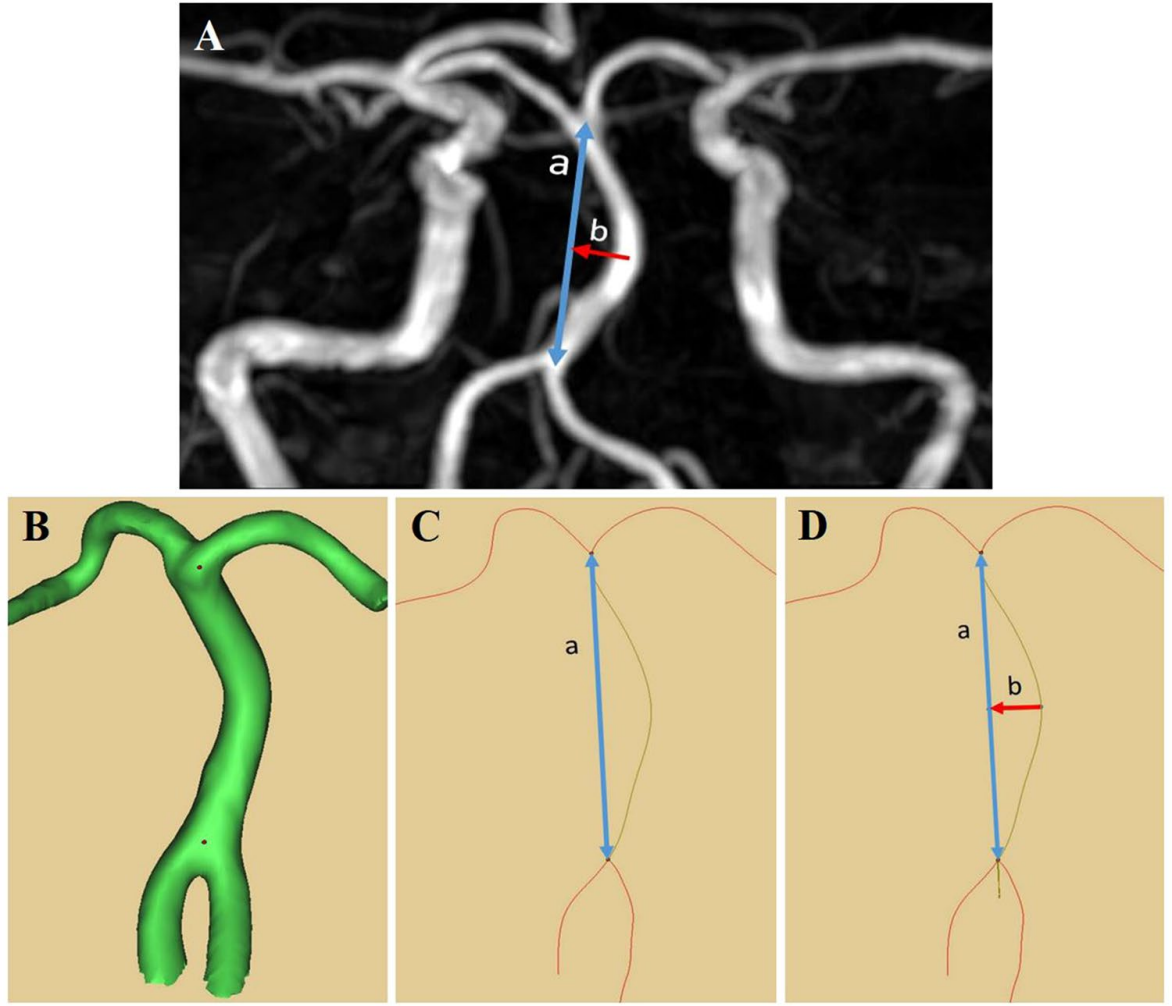
The geometry of the BA has been measured previously using magnetic resonance images **(A)**, including the basilar artery length (a) and basilar artery bending length (b). Modification of method is used in order to improve the quantitative analysis using the MIMICS software **(B-D)**.

Table 1 shows the comparative results for all demographic variables between the 2 study populations. There was no statistically significant difference in age and follow-up time between population with non-dominant VA and those with VA dominance (p = 0.44 and p = 0.93, respectively); however, there were significant differences in gender, height, and mass between the 2 groups. The mean height of the population with VA dominance is greater (160.8 ± 8.2 centimeters vs. 157 ± 6.9 centimeters). Besides, subjects with VA dominance had greater rates of hyperlipidemia.

**Table 1:** Study population characteristics at baseline.

	Non-dominant VA	VA dominance	p value
	n = 46	n = 108	
**General demographic**			
Gender (male, %)	13 (28.3%)	51 (47.2%)	0.03*
Age (years)	60 ± 12.3	59,6 ± 10.6	0.44
Height (cm)	157 ± 6.9	160.8 ± 8.2	0.02*
Mass (kg)	59.4 ± 9.8	63.9 ± 10,7	0.02*
**Risk factors**			
Hyperlipidemia	9 (19.6%)	9 (8.3%)	<0.05*
Smoking	5 (10.9%)	7 (6.5%)	0.35
Diabetes mellitus	10 (21.7%)	19 (17.6%)	0.55
Cardiac disease	8 (17.4%)	13 (12%)	0.38
Previous stroke	12 (26.1%)	36 (33.3%)	0.37
Hypertension	29 (63%)	51 (47.2%)	0.07
Follow-up time (months)	123.4 ± 14.9	123.7 ± 16.7	0.93

VA, vertebral artery.

The values are presented as the mean ± standard deviation or percentage, as appropriate.

The p values are from the independent t-test or chi-square test, as appropriate.

*Indicates p < 0.05.

A mixed model ANOVA was conducted to investigate the impact of aging and VA dominance on the change of BABL after 123.6 ± 16.2 months (Table 2). There was not a significant main effect of aging, F (1,152) = 3.28, p = 0.07. However, there was a significant main effect of VA dominance on the change of BABL after about 10 years, F (1,152) = 39.78, p < 0.01. The bending of the BA was more pronounced in patients with VA dominance than in patients without it (6.4 ± 0.3 mm vs. 2.4 ± 0.5 mm, respectively). On the other hand, there was no significant interaction between aging and VA dominance, F (1,152) = 3.16, p = 0.08.

**Table 2:** Mixed model ANOVA results of the basilar artery bending length change after 123.6 ± 16.2 months (within-group factor: aging; between-group factor: vertebral artery dominance).

Dependent variable	Source of variation	F ratio	p value	Comparison*
BABL	Aging	3.28	0.07	
	VA Dominance	39.78	<0.01	VAD > non-VAD
	Interaction	3.16	0.08	

BABL, basilar artery bending length; VAD, vertebral artery dominance group.

* The comparison was performed for the mean value of 2 categories, where significant differences were found.

We also performed the mixed model ANOVA to investigate the impact of aging and VA dominance on the change of BAL during the same period of time (Table 3). There was a significant main effect of aging on the change of BAL, F (1,152) = 6.64, p = 0.01 (27.1 ± 0.4 mm for baseline vs. 27.5 ± 0.4 mm for the follow-up after 10-year). However, there was not a significant main effect of VA dominance, F (1,152) = 1.76, p = 0.19. Besides, there was not a significant interaction between aging and VA dominance, F (1,152) = 0.58, p = 0.45.

**Table 3:** Mixed model ANOVA results of the basilar artery length change after 123.6 ± 16.2 months (within-group factor: aging; between-group factor: vertebral artery dominance).

Dependent variable	Source of variation	F ratio	p value	Comparison*
BAL	Aging	6.64	0.01	B < F
	VA Dominance	1.76	0.19	
	Interaction	0.58	0.45	

BAL, basilar artery length; B, Baseline measurement; F, Follow-up measurement.

* The comparison was performed for the mean value of 2 categories, where significant differences were found.

**Figure 4:**
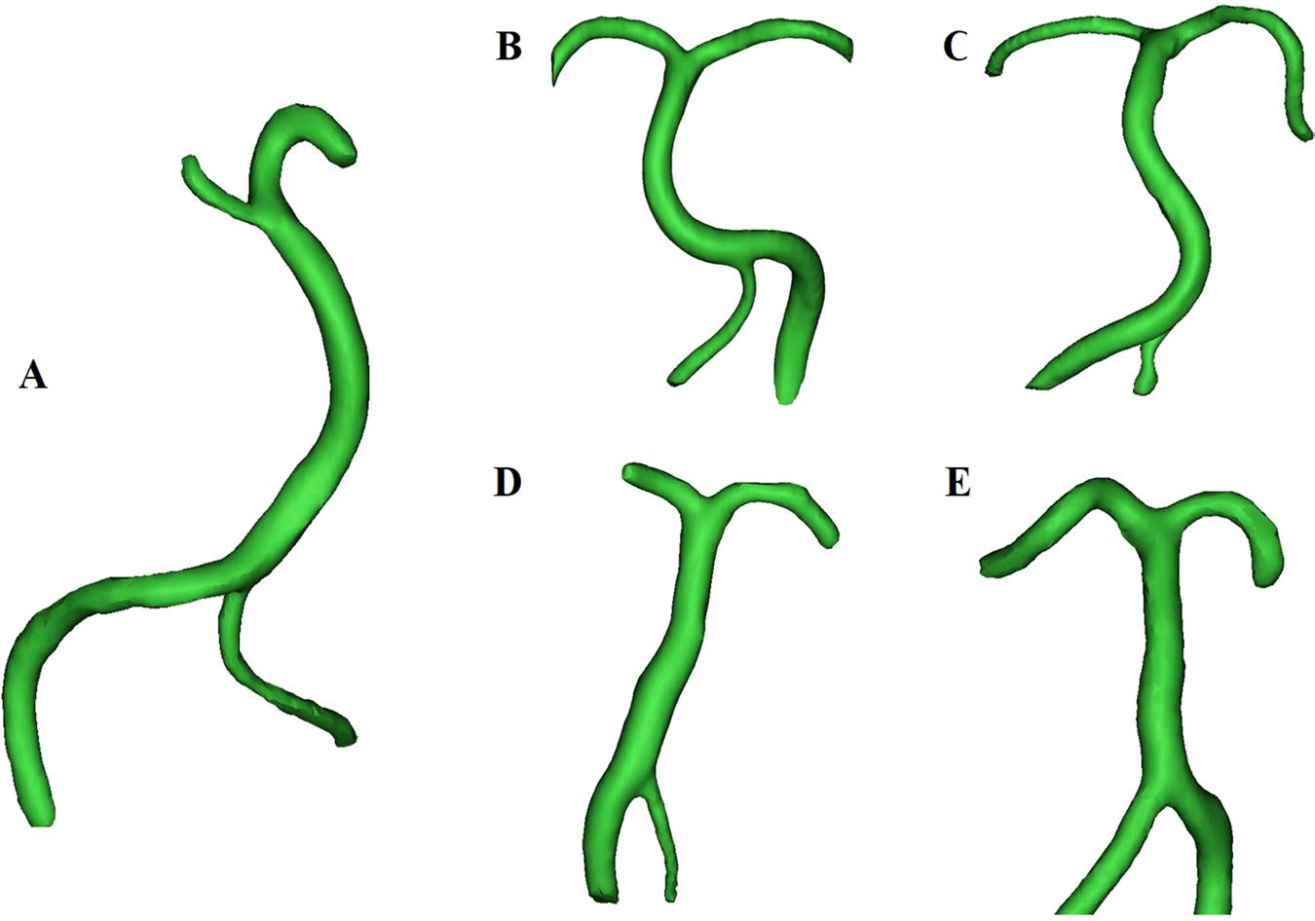
The geometric change of the BA was observed from the anteroposterior view. C-shaped deformation (**A**), J-shaped (**B**), S-shaped (**C**), diagonal line-shaped (**D**), and no deformation or straight (**E**).

Table 4 reports the results of chi-square test on the directional relationship between the dominant VA and BA bending. Findings indicated that the BA bending side significantly associated with the VA dominant side (p < 0.01), with most subjects (77/108; 71.3%) had an opposite directional relationship between the dominant VA and BA bending. Within the 108 cases of the VA dominance group, the dominant VA side was more frequent on the left (69.4%). The geometric changes of the BA, as shown in Fig. 4, were as follow: C-shaped deformation (n = 67), J-shaped (n = 15), S-shaped (n = 6), diagonal line-shaped (n = 5), and no deformation or straight (n = 15).

**Table 4:** Chi-square test on the directional relationship between the dominant vertebral artery and basilar artery bending.

		Basilar artery bending side			Total
		Right	Left	Straight	
Dominant side	Right	8 (7.4%)	21 (19.4%)	4 (3.7%)	33 (30.6%)
	Left	56 (51.9%)	8 (7.4%)	11 (10.2%)	75 (69.4%)

df = 2; χ2 = 33.886; p < 0.01.

Values are presented as number (percentage).

## Discussion

In the present study, we investigate the impact of aging and VA dominance on the change of BA geometry after a decade of life. We found that there was no significant interaction effect of aging and VA dominance on the change of both BABL and BAL. This result permitted to investigate separately whether individual aging or VA dominance, had an effect on the change of BA geometry over time. We found that there was not a significant main effect of aging on the change of BABL after a decade of life. This result is consistent with the report of Nishikata *et al*.^9^ that BABL was not correlated with age. On the other hand, our study identified that there was a significant main effect of VA dominance on the change of BABL after 10 years, with the bending of the BA was more pronounced in patients with VA dominance than in patients without it. This result supports the hypothesis that the asymmetrical diameter of the 2 VAs is the main contributor to BA bending over time^13^. In addition, the results of the previous study by Zhu *et al*.^13,21^ also supported the opinion that VA dominance results in BA bending. VA dominance can lead to asymmetry of vertebrobasilar junction blood flow and may result in mechanical change, causing BA curvature, more asymmetry of bilateral VA diameter, and a greater difference in the impact force of BA blood flow; ultimately, an increase in BABL may result^11,12^.

Variations in the position and length of the BA can be attributed to aging and hemodynamic factors^22^. We found that there was a significant main effect of aging on the change of BAL after 10 years. By contrast, there was not a significant main effect of VA dominance on the change of BAL during the same period of time. This result suggests that progressive BA elongation is related to vascular remodeling during aging. Nishikata *et al*.^9^ also found that BAL and age correlated. Our longitudinal study, based on changes in individual persons, corroborated evidence that longitudinal lengthening of the BA depends on aging.

In our study, the dominant VA side was more frequent on the left, which is consistent with numerous previous report^13^. Several studies have investigated flow-dependent remodelling^23–25^; if these hemodynamic forces are chronically altered, there might be a morphological or structural adaptation of the vessel to minimize their impact on the arterial wall, including changes in the wall thickness and caliber, as well as arterial geometry. In this study, there is a significant association between the dominant VA side and BA bending side, with most subjects had an opposite directional relationship between the dominant VA and BA bending. This result also supports the conclusion that VA dominance resulted in BA bending. Indeed, the asymmetric inflow of VA dominance results in changes of the flow force along the proximal portion of the BA, which can cause specific morphological deformation in the VBA system^14^. In the present study, the most frequent morphology change of the BA is C-Shaped. A similar result was obtained by Hong *et al*.^13^. However, the geometric change of BA with aging is multiform. Further studies are needed to improve our understanding of the mechanism underlying these age-related changes and other factors affecting such as mechanical wall shear stress and plaque progression.

We acknowledge that this study has limitations. First, it is a retrospective study, with the inherent possibility of selection bias. Second, because the cerebral MRAs were performed for clinical indications, the included subjects were more likely than the general population to have vascular risks. However, the inclusion criteria of subjects with only normal VBA systems may have helped diminish this effect. Third, as our study was not intended to be an epidemiological study, our patients do not necessarily represent the characteristics of the general population. Fourth, there were significant gender ratio differences between the 2 groups in our study. However, to the best of our knowledge, there is no report relating to gender difference and VA dominance. A study by Deng *et al*.^26^ found that there is no significant gender difference was found in height or the position of the BA. In addition, in the study by Hong *et al*.^13^, gender was not associated with BA curvature. Fifth, measurement of BAL and BABL, while reliable and simply determined, may not be the optimal measurement to evaluate vessel tortuosity, as it does not examine other geometric factors that may affect flow dynamics, such as BA angulation, angles of the inflow feeding VA, and PCA outflow. Finally, we did not perform high-resolution MRA or flow dynamic analysis to determine the intraluminal status and blood flow characteristics of the BA.

## Conclusion

Our longitudinal study, based on changes in individual persons, supported the hypothesis that bending of the BA depends on the dominance of the VA, whereas increased length of the BA depends on aging.

## Supplementary information

Supplementary information.
