# Supplementary material for: Change in basilar artery length and bending according to aging and vertebral artery dominance: A longitudinal study
Source: Sci Rep. 2020 Jun 1;10:8904. doi: 10.1038/s41598-020-65682-x (PMC7264222; doi:10.1038/s41598-020-65682-x)
Supplement: Supplementary file 1 — Supplementary information. [file 41598_2020_65682_MOESM1_ESM.docx]

**Change in basilar artery length and bending according to aging and vertebral artery dominance: A longitudinal study**

Minh Tri Ngo, MD.^1^, Hyo Sung Kwak, M.D., Ph.D.^1^, Gyung Ho Chung. M.D., Ph.D.^1^.

**Table** Univariate analysis of cardiovascular risk factors linking basilar artery geometric parameter

|  | BABL | | BAL | |
| --- | --- | --- | --- | --- |
|  | ANOVA | | ANOVA | |
|  | *F* value | *p* value | *F* value | *p* value |
| Hyperlipidemia | 0.619 | 0.433 | 0.000 | 0.993 |
| Smoking | 0.07 | 0.935 | 1.726 | .0191 |
| Diabetes | 6.806 | 0.010 | 0.878 | 0.350 |
| Cardiac disease | 1.227 | 0.270 | 0.602 | 0.439 |
| Stroke | 0.545 | 0.462 | 0.002 | 0.969 |
| Hypertension | 1.667 | 0.199 | 0.985 | 0.323 |

ANOVA, analysis of variance; BABL, basilar artery bending length, BAL basilar artery length.
BABL and BAL were introduced as continuous variables and were measured at baseline.
*Indicates *p* < 0.05.
